# Advances in Mental Health and Well-Being in Children (2nd Edition)

**DOI:** 10.3390/children13101383

**Published:** 2026-10-12

**Authors:** Cláudia Carmo, Cristina Nunes

**Affiliations:** University Research Centre in Psychology (CUIP), Universidade do Algarve, 8005-139 Faro, Portugal; csnunes@ualg.pt

The promotion of mental health and well-being in children and adolescents is currently one of the key priorities in research, clinical practice, and public policy [1,2]. The recognition that a significant proportion of mental disorders begin during childhood and adolescence, together with evidence that early experiences shape developmental trajectories across the lifespan, reinforces the importance of prevention, early detection, and the promotion of contexts that foster healthy psychological development.

Rather than merely preventing illness, it is increasingly necessary to create conditions that enable children and young people to develop the competencies, relationships, and resources needed to face the challenges inherent in their developmental pathways, in line with international recommendations on the reorganisation of mental health systems for these age groups [3].

Over the past decades, research has consistently shown that mental health results from the dynamic interaction between biological, psychological, family, school, social, and cultural factors. This ecological and developmental perspective has contributed to a paradigm shift, moving away from models predominantly focused on psychopathology towards approaches that also emphasise protective factors, resilience, competence promotion, and developmental potential in children and adolescents [1,2]. In the same line, there has been a growing investment in evidence-based interventions, innovation in psychological assessment, and the implementation of programmes across multiple contexts (families, schools, health services, and community settings), as highlighted in recent reviews of child and adolescent mental health care models [3].

This is the context in which this Special Issue on Mental Health and Well-Being in Children is situated. Despite the diversity of contexts, methodologies, and populations studied, the papers included in this issue converge on a shared perspective: to better understand the processes that promote mental health and to identify effective strategies for its promotion throughout development.

In a first approach, the set of studies highlights the central role of the family as a privileged developmental context. Research on the intergenerational transmission of perfectionism and parenting programmes underscores the influence of parenting practices, temperament characteristics, and the quality of family relationships in shaping children’s emotional and behavioural regulation. These contributions highlight that the promotion of mental health often begins in the child’s closest environments, and that strengthening parenting skills is a key strategy for enhancing protective factors from the earliest years of life.

The published articles also highlight the importance of a preventive approach grounded in competence promotion rather than solely in the reduction in risk factors. Interventions aimed at developing socioemotional skills in school settings, psychological support for adolescents with type 1 diabetes, and cognitive–behavioural therapy-based educational strategies illustrate the potential of early interventions to promote well-being, strengthen individual resources, and foster more adaptive developmental trajectories. These studies reinforce the need to invest in the promotion of mental health during childhood and adolescence as one of the most effective strategies for improving quality of life across the lifespan [1].

The diversity of the studies presented highlights the complexity of the factors influencing psychological development. Self-regulation, resilience, self-efficacy, happiness, and problem-solving skills emerge as important protective resources, while contemporary challenges such as social media use, or adverse experiences such as institutionalisation, illustrate the multiplicity of factors that can shape developmental pathways. This diversity reinforces the need for ecological and multidimensional approaches, consistent with mental health research [2].

Psychological assessment constitutes another key focus of this Special Issue. The development and validation of new instruments for assessing autism spectrum behaviours, as well as reflections on projective construction techniques in the digital era, highlight that scientific progress also depends on the availability of rigorous, culturally sensitive tools adapted to the technological and social innovations characterising contemporary psychological practice.

This volume also includes contributions from different disciplinary fields, reflecting the growing openness of research to interdisciplinary collaboration. The review on the potential of medicinal plants in child mental health is an example of this diversity of perspectives and underscores that therapeutic innovation must be accompanied by a critical evaluation of the effectiveness of different approaches.

Taken together, the published articles illustrate an important evolution in child and adolescent mental health research, highlighting international priorities for narrowing the gap between needs and responses in mental health care [2]. There is also a growing integration between basic research, instrument development and validation, evaluation of intervention effectiveness, and the implementation of programmes in real-world settings.

This issue invites reflection on the challenges that continue to shape this field. It is essential to deepen knowledge of the mechanisms underlying developmental trajectories of adaptation and vulnerability, strengthen the evaluation of implementation and sustainability of interventions in real-world contexts, promote robust longitudinal research, and ensure that scientific knowledge is effectively translated into public policies and professional practices capable of reducing inequalities and promoting psychological well-being from early childhood.

Rather than simply bringing together a set of studies on child and adolescent mental health, this Special Issue reflects a growing scientific field characterised by the integration of multiple disciplines, methodologies, and intervention contexts. The diversity of contributions published in this issue demonstrates that understanding and promoting mental health requires integrated responses, sustained by continuous collaboration between research, clinical practice, education, and public health [2,3].

We express our sincere gratitude to all authors, reviewers, and readers for their contribution to this issue. The scientific rigour, availability, and commitment of all involved were essential to the quality of this Special Issue. We hope that the published papers will contribute to advancing scientific knowledge, stimulating new lines of research, and strengthening dialogue between researchers, clinicians, educators, and policymakers, inspiring increasingly effective practices in promoting the mental health and well-being of children and adolescents.
